# Asymmetries observed in Saturn's magnetopause geometry

**DOI:** 10.1002/2015GL065477

**Published:** 2015-09-03

**Authors:** N. M. Pilkington, N. Achilleos, C. S. Arridge, P. Guio, A. Masters, L. C. Ray, N. Sergis, M. F. Thomsen, A. J. Coates, M. K. Dougherty

**Affiliations:** ^1^ Atmospheric Physics Laboratory, Department of Physics and AstronomyUniversity College LondonLondonUK; ^2^ Centre for Planetary Sciences at UCL/BirkbeckLondonUK; ^3^ Physics DepartmentLancaster UniversityLancasterUK; ^4^ Blackett LaboratoryImperial College LondonLondonUK; ^5^ Office of Space Research and TechnologyAcademy of AthensAthensGreece; ^6^ Planetary Science InstituteTucsonArizonaUSA; ^7^ Mullard Space Science Laboratory, Department of Space and Climate PhysicsUniversity College LondonDorkingUK

**Keywords:** Saturn, magnetopause, asymmetry, season, magnetosphere, plasma

## Abstract

For over 10 years, the Cassini spacecraft has patrolled Saturn's magnetosphere and observed its magnetopause boundary over a wide range of prevailing solar wind and interior plasma conditions. We now have data that enable us to resolve a significant dawn‐dusk asymmetry and find that the magnetosphere extends farther from the planet on the dawnside of the planet by 7 ± 1%. In addition, an opposing dawn‐dusk asymmetry in the suprathermal plasma pressure adjacent to the magnetopause has been observed. This probably acts to reduce the size asymmetry and may explain the discrepancy between the degree of asymmetry found here and a similar asymmetry found by Kivelson and Jia (2014) using MHD simulations. Finally, these observations sample a wide range of season, allowing the “intrinsic” polar flattening (14 ± 1%) caused by the magnetodisc to be separated from the seasonally induced north‐south asymmetry in the magnetopause shape found theoretically (5 ± 1% when the planet's magnetic dipole is tilted away from the Sun by 10–17°).

## Key Points

- Saturn's magnetosphere extends farther at dawn than dusk by  7%
- The degree of asymmetry departs from MHD predictions, likely due to the suprathermal plasma pressure
- An intrinsic polar flattening of  14% is found with an additional seasonally induced component

## 1 Introduction

Plasma plays an important role in shaping Saturn's magnetosphere. Plumes of water ice grains and molecules are ejected from Enceladus [e.g., *Dougherty et al.*, 2006; *Porco et al.*, 2006], and a fraction of these are ionized and picked up by the magnetic field. This newly formed plasma is then accelerated from the Keplerian velocity of the moon up to the typically subcorotational flow velocity of the ambient plasma, ultimately via ion‐neutral collisions in the ionosphere conveyed to the more distant parts of the flux tube via a **j** × **B** force.

The thermal component of this plasma is largely equatorially confined due to centrifugal forces and forms an extended magnetodisc structure [e.g., *Arridge et al.*, 2007], which stretches all the way out to the dayside magnetopause. The pressure associated with the magnetodisc is large enough to inflate the equatorial magnetosphere significantly more than the high‐latitude magnetosphere, and this results in polar flattening of the magnetopause boundary [*Pilkington et al.*, 2014]. Unlike the equatorially confined thermal plasma, energetic plasma is ubiquitous within Saturn's magnetosphere [e.g., *Krimigis et al.*, 1982, 2005] and can extend to high latitudes. At the magnetopause, the pressure associated with the suprathermal (≥30keV) component is comparable to the effective pressure of the magnetic field [*Kanani et al.*, 2010], and *Pilkington et al.* [2015] found that the suprathermal plasma pressure can strongly affect the location of the magnetopause.

Here we will extend the analysis of *Pilkington et al.* [2015] and consider the effect of the subcorotational cold plasma population and the suprathermal plasma population on the global morphology of Saturn's magnetopause. These observations span almost a third of a Kronian year. Theoretical studies by *Maurice et al.* [1996] and *Hansen et al.* [2005] found that the geometry of the magnetopause changes significantly with planetary season. In particular, a north‐south asymmetry in the distance between the planet and the magnetopause is introduced when there is a significant tilt between the magnetic dipole and the incoming solar wind direction in the Kronocentric Solar Magnetospheric (KSM) coordinate system. In this system, the *X*_KSM_ axis points from the planet toward the Sun, the *Z*_KSM_ axis is such that the magnetic dipole is contained within the *X*_KSM_‐*Z*_KSM_ plane, and the *Y*_KSM_ points toward dusk to complete the right‐handed set. As such, this asymmetry will also be quantified in terms of the apparent polar flattening/inflation imposed by the orientation of the planetary dipole with respect to the solar wind flow direction, which changes with season.

## 2 Fitting to Magnetopause Observations

**Figure 1:**
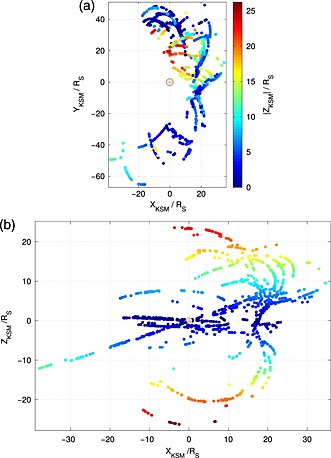
The distribution of magnetopause crossings in the KSM coordinate system, colored by their *Z*_KSM_ coordinate with the planet at the origin.

As detailed by *Pilkington et al.* [2015], an extensive database of in situ magnetopause crossings observed at Saturn has been compiled, utilizing data collected over the majority of the Cassini mission timeline. Magnetic field data from the Cassini fluxgate magnetometer (MAG) [*Dougherty et al.*, 2002] and electron plasma data from the Cassini Plasma Spectrometer (CAPS‐ELS) [*Young et al.*, 2004] have been used to facilitate the identification of magnetopause crossings. This data set samples most regions of the dayside magnetopause (Figure 1) over a period of 8 years, in contrast to previous studies which were necessarily restricted to smaller regions in space and shorter time periods [e.g., *Slavin et al.*, 1983; *Arridge et al.*, 2006; *Kanani et al.*, 2010]. Specifically, this study covers from 28 June 2004 (just prior to Saturn Orbit Insertion) through to 29 October 2010, and from 13 May 2012 to 8 February 2013. The orbits between 29 October 2010 and 13 May 2012 sampled a very similar region to that sampled in the preceding year: the equatorial region on the duskside of the planet. As such, they would not have contributed much to this study. Owing to the time‐consuming nature of the analysis, the orbits that took place after 13 May 2012 were prioritized during which the high‐latitude southern hemisphere was sampled.

To isolate the impact of internal plasma dynamics on magnetopause morphology, it is first necessary to remove the effects of external variability by correcting for changing solar wind dynamic pressure. In the absence of a dedicated upstream pressure monitor, a Newtonian approximation to supersonic flow about a body [*Petrinec and Russell*, 1997] was used to relate the upstream dynamic pressure to internal pressure sources measured in situ. Specifically, the effective solar wind pressure acting normal to the model magnetopause surface at the location of each crossing is balanced against the interior magnetic, thermal electron, thermal ion, and suprathermal ion pressures derived from MAG, CAPS‐ELS, the CAPS ion mass spectrometer, and the Magnetospheric Imaging Instrument (MIMI) [*Krimigis et al.*, 2004; *Sergis et al.*, 2009], respectively.

Here the empirical magnetopause model of *Shue et al.* [1997] is used,

(1) $$r=r_{0}{\left(\frac{2}{1+\cos\theta }\right)}^{K}$$

(2) $$r_{0}=a_{1}D_{P}^{-a_{2}}$$

(3) $$K=a_{3}+a_{4}D_{P}$$

where *r* and *θ* are polar coordinates that describe a point on the magnetopause. The parameter *r* denotes the distance from the planet's center (the origin), and *θ* is the angle between the planet‐Sun line and the position of the point on the magnetopause with respect to the origin. The parameter *r*_0_ is the subsolar standoff distance and *K* is the “flaring parameter” which controls the downstream shape of the magnetopause surface. If *K* = 0.5, the magnetosphere is “open” with a constant tail radius. Above this value, the model shape expands with distance from the planet, and below this value the magnetosphere is “closed.” Coefficients *a*_1 − 4_ control, respectively, (1) the size scale of the magnetosphere; (2) its response to changes in solar wind dynamic pressure; (3) the nominal downstream flaring of the magnetosphere; and finally, (4) the effect of dynamic pressure on magnetopause flaring.

This model is rotationally symmetric about the planet‐Sun line, but a significant degree of polar flattening has been observed by *Pilkington et al.* [2014]. Hence, this model is modified in order to incorporate polar flattening by scaling the surface along the north‐south direction, *Z*, by a factor E where E<1. Hence, the axisymmetric surface described by equations (1), (2), (3) can be transformed into a “flattened” surface as follows:

(4) $$\left(\begin{array}{c} x^{\prime }\\ y^{\prime }\\ z^{\prime } \end{array}\right)=\left(\begin{array}{ccc} 1 & 0 & 0\\ 0 & 1 & 0\\ 0 & 0 & E \end{array}\right)\left(\begin{array}{c} x\\ y\\ z \end{array}\right)$$

where x=rcosθ, y=rsinθcosϕ, z=rsinθsinϕ, and *ϕ* is the angle between the projection of *r* onto the *Y*_KSM_‐*Z*_KSM_ plane and the *Y*_KSM_ axis.

This model is fitted iteratively to the magnetopause observations by minimizing the root‐mean‐square (RMS) distance between each observed magnetopause crossing and the model surface, constructed separately at the dynamic pressure estimated for each crossing assuming pressure balance. The distance between each crossings and each model surface is calculated near exactly by solving a set of four equations numerically as outlined by *Pilkington et al.*[2015]. This effectively provides an exact solution since these equations can be solved to an arbitrary degree of accuracy, here the calculation is iterated until it converges to a distance smaller than 10^−6^ *R*_*S*_. The *Matlab Optimization Toolbox* implementation of the interior‐point algorithm of *Waltz et al.* [2005] is employed as the local solver, and the global optimization algorithm of *Ugray et al.* [2007] is used to efficiently sample parameter space in order to maximize the chance of converging on the global minimum.

## 3 The Asymmetries

### 3.1 Dawn‐Dusk

In the first instance, to determine if a dawn‐dusk asymmetry could be present, all model coefficients besides those that control the extent of tail flaring, *K*, were fixed to the values found by *Pilkington et al.* [2015]. Separate fits were then made to crossings in the noon‐dawn and noon‐dusk sectors. One could allow all parameters to vary but the degree of polar flattening on the dawnside of the planet is ill‐constrained due to the lack of high‐latitude prenoon crossings. This could affect the other parameters as, away from the equator, a flatter, more flared surface and a less flattened but less flared surface could fit any given magnetopause crossing equally well.

The best fitting values of *K* in each case implied that there is a statistically significant difference in the tail flaring on the dawn and dusk flanks with the magnetopause extending farther on the dawn flank. However, there are several problems with this methodology. First, imposing a different degree of flaring in this way leads to a discontinuous surface at the poles. Also, as pointed out by *Petrinec and Russell* [1995] and *Joy et al.*[2002], empirical models can be biased by the simple fact that the magnetopause is only sampled along the spacecraft trajectory for the solar wind conditions and magnetospheric configuration present at the time of the crossing.

To minimize the impact of observational bias, the data were reduced in order to provide equal spacecraft sampling on either side of the planet. It can be seen in Figure 1 that observations of the dawn magnetopause extend farther in the *X*_KSM_ and *Y*_KSM_ directions than the observations of the magnetopause at dusk, and the high‐latitude observations lie on the duskside of the planet only. As a result, the trajectory of the spacecraft has been considered and crossings that lie in a region that has not been adequately sampled on the opposite side of the planet have been removed. Specifically, crossings are accepted that satisfy the conditions |*Z*_KSM_|≤10 and −18≤*X*_KSM_≤24, the latter of which also accounts for potential biases in *Y*_KSM_ due to the trajectory of the spacecraft. This left 989 magnetopause crossings with which to proceed with the analysis. The mean magnetopause position found in previous studies is still captured despite these restrictions, e.g., *Achilleos et al.* [2008] and *Pilkington et al.* [2015].

Since the observations on the dawnside and duskside of the planet are typically separated in time by years, long‐terms trends in the dynamic pressure due to the solar cycle, for example, could potentially affect the results of this study. To ensure that the upstream conditions were similar when the observations on each side of the magnetopause were made, the Kolmogorov‐Smirnov test [*Massey*, 1951] has been used. This tests the null hypothesis (the hypothesis accepted unless contradicted by the results of the test) that two independent random samples are drawn from the same underlying continuous population. Here it is used to check that the dynamic pressure is equally distributed for observations made on both sides of the planet. The null hypothesis could not be rejected at the 1*σ* significance level, indicating that the probability that the dynamic pressure distributions are significantly different is less than 69.1%, the lowest level of significance usually considered. This indicates that systematic differences in the pressure distributions on either side of the planet are insignificant.

Throughout the period that this study encompasses, the spacecraft spent approximately 3 years sampling the dawnside of the planet, but one of these years was primarily spent traversing the magnetotail during which the magnetopause could not be observed. It spent approximately 5 years sampling the duskside of the planet, so naturally there were more observations of the magnetopause here. If an empirical model were fitted to the reduced set of magnetopause crossings outlined above, the fit would be artificially weighted to the dusk magnetopause as a result. Such a weighting is removed by randomly sampling the data on the duskside to balance the number of crossings on either side. A technique known as “stratified sampling” is used to mirror the local time distribution of crossings to prevent artificial weighting to “overpopulated” local time sectors.

The procedure is as follows: the dawn crossings are separated into local time bins with a width of 1 h and the percentage of crossings within each bin is calculated. The probability of a crossing occurring within each local time bin on the dawnside of the planet is then calculated and imposed on the random sample drawn from crossings on the duskside. In any cases where there are fewer crossings on the duskside in a particular mirrored local time bin, the dawn crossings in that particular bin are randomly sampled instead. The end result is an equivalent number of dawn and dusk crossings equally dispersed in terms of local time, to which the model is fitted. 716 magnetopause crossings remained in total.

The functional form of the flaring parameter has been modified to introduce a dawn‐dusk asymmetry,

(5) $$K=a_{3}+a_{4}D_{P}+a_{5}\cos\phi$$

where *ϕ* is the angle between the *Y*_KSM_ axis and the projection of the radial vector onto the *Y*_KSM_−*Z*_KSM_ plane, and *a*_5_ is a free parameter found by fitting the empirical surface to the data. The magnitude of *a*_5_ hence controls the size of the dawn‐dusk asymmetry and cosϕ increases the tail flaring on one side of the planet and reduces it on the opposite side depending on the sign of *a*_5_ with a smooth transition between them. As such, the asymmetry in terms of the flaring parameter will be of magnitude 2*a*_5_ at the equator in the *X*_KSM_−*Y*_KSM_ plane.

Repeating the fitting using (5) and the reduced data set outlined above confirms that the magnetopause extends farther on the dawn flank. Since the crossings are sampled randomly, this procedure is repeated 100 times in order to ensure that the results are insensitive to which crossings are selected. This yields a median value of *a*_5_=−0.024 ± 0.007 and reduced the RMS residual by ∼10% relative to a fit without the asymmetry term in equation (5). For the sake of comparison, *a*_3_=0.688 ± 0.006. A significance test, such as the *F* test, can be used to test the hypothesis that adding the extra free parameter significantly improves the predictive power of the model and simply relies on the improvement in the model prediction being large enough to outweigh the cost of adding extra free parameters. Employing this test, the probability that this improvement was caused by random scatter in the data is negligible.

This asymmetry may be caused physically by the intrinsic asymmetry in plasma flow around the planet with respect to the direction of solar wind flow. Plasma generally flows in the same direction as the planet rotates, so it flows against the direction of the solar wind at dawn and in the same direction as the solar wind at dusk. Hence, the magnetopause may be pushed farther from the planet at dawn than at dusk.

**Figure 2:**
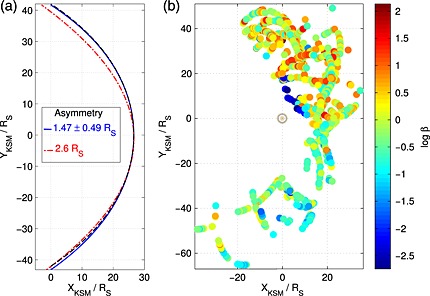
(a) Comparison of the empirical dawn‐dusk asymmetry from this work (blue solid) to that derived from the MHD model of Kivelson and Jia [2014] (red dash‐dotted) and a symmetric magnetopause model (black dashed) constructed at the same standoff distance. (b) The spatial distribution of magnetopause crossings colored by *β*, the ratio of the suprathermal plasma pressure to the magnetic pressure. In general, *β* is several times larger for crossings at dusk than at dawn and so reduces the extent of the empirical size asymmetry somewhat compared to the MHD results. The cluster of very low *β* crossings on the duskside of the planet is likely to map to the cusp region.

MHD simulations by *Kivelson and Jia* [2014] predict a mean asymmetry of 2.6*R*_S_ at the terminator plane compared to our empirically derived asymmetry of 1.47 ± 0.49*R*_S_ when the surface is constructed at the same standoff distance (26.61*R*_S_). The surfaces have been compared at the same standoff distance rather than the same dynamic pressure, because we have found that the asymmetry is insensitive to changes in the dynamic pressure within the limitations of the data used in this study, whereas the absolute size of the asymmetry changes with system size. It should be noted though that the empirical model predicts that a dynamic pressure of 0.011 ± 0.01 nPa is necessary for Saturn's magnetosphere to have a standoff distance of 26.61*R*_S_, which is significantly different from the dynamic pressure used in the simulation of 0.013 nPa. A comparison between the MHD and the empirical surface is shown in Figure 2a.

The largest discrepancy between these surfaces is at dusk, where the empirical surface lies farther from the planet than the theoretical MHD surface. The discrepancy between these results can be explained by considering the effect that suprathermal plasma has on the magnetopause. *Pilkington et al.* [2015] found that the size of the magnetosphere is strongly correlated with *β*, the ratio of suprathermal pressure to the magnetic pressure, just inside the magnetopause. The plasma *β* indicates the control that plasma has on the system. Plasma is confined by the magnetic field to varying degrees depending on the particle energy, so for the system to change in size as a result of a sudden influx of hot plasma, for example, [e.g., *Krimigis et al.*, 2007], the plasma pressure must be sufficient to overcome the constraining effect of the magnetic field and change the field structure across both local and global scales.

An asymmetry in the suprathermal plasma pressure has been observed and is shown in Figure 2b in terms of the suprathermal plasma *β*. This is in the opposite sense to the aforementioned asymmetry in the distance from the planet to the magnetopause at dawn and dusk. As such, the suprathermal plasma can perturb the magnetopause more at dusk than at dawn, pushing the magnetopause farther out at dusk than where it would otherwise be located. A similar asymmetry is seen in the ring current (N. Sergis, private communication, 2015). The cause of this asymmetry is unknown but may be caused by the change in the flux tube configuration as they move around the planet. As expanded flux tubes in the dawn sector flow around the planet through noon, they are forced into a smaller volume because they move from the spacious magnetotail and into the dayside magnetosphere [*Kivelson and Southwood*, 2005; *Delamere and Bagenal*, 2013]. Hence, one may expect the plasma within them to be heated adiabatically. However, magnetic field lines and hence flux tubes follow a lagging configuration in the sense of corotation [*Khurana and Schwarzl*, 2005]. As they flow through noon and into the dusk sector, they expand again somewhat, but not to the same size as they were at dawn since, at dawn, they are free to drape all the way back into the magnetotail and can occupy a much larger volume. But at dusk the lack of magnetic field bend back causes flux tubes to encounter the dayside magnetopause, which restricts their expansion. As such, the plasma within these flux tubes remains compressed and energized and causes the flux tubes to expand and push the magnetopause farther out than it would be located in the absence of this effect.

This effect is not present in the results of *Kivelson and Jia* [2014] as the suprathermal plasma population is not included in the MHD simulations. As a result, the size asymmetry in the simulations appears larger than the empirically observed asymmetry since there is no suprathermal plasma pressure to counter it. Also, note that the orbital motion of the planet causes the incoming solar wind to be rotated by ∼1.4° out of the *X*_KSM_‐*Z*_KSM_ plane such that the solar wind is preferentially flowing into the dawnside of the planet. This may also be expected to produce a small dawn‐dusk asymmetry in the opposite sense to that detected here and probably reduces the asymmetry somewhat. *Kivelson and Jia* [2014] assume that the solar wind flows along the Saturn‐Sun line or the −*X*_KSM_ direction. If the aberration of flow does, indeed, cause a small asymmetry, then including it in the simulation would partially reduce the discrepancy between the observations and the MHD prediction.

### 3.2 Seasonal Variability

*Maurice et al.* [1996] constructed a semiempirical magnetic field model of Saturn consisting of planetary dipole, current sheet, and magnetopause contributions, partly based on data taken by *Voyager 1* and *Voyager 2*. They were able to derive the shape of the magnetopause from this information assuming a fixed solar wind dynamic pressure. They found that the magnetopause geometry changed with planetary season due to variations in the angle between the magnetic dipole and the *Z*_KSM_ axis, *λ*. When their surface is recast into the KSM coordinate system, there is a significant difference in its geometry in opposite hemispheres in the presence of a significant dipole tilt. The smallest dipole tilt tested by *Maurice et al.* [1996] was 15°, but the effect is probably present at smaller values than this as a gradual deflection, increasing with *λ*. Specifically, their surface is flatter in the north and more elongated in the south under conditions similar to those when Cassini arrived at Saturn (*λ*=–26.7°). Similar results were obtained by *Hansen et al.* [2005] through MHD simulations of the Kronian magnetosphere. Observations of such seasonal changes can be used to separate this effect from intrinsic flattening arising from the disklike nature of the obstacle to solar wind flow due to the presence of the magnetodisc, which preferentially inflates the low‐latitude magnetosphere.

Such a study is hampered by the fact that the high‐latitude observations utilized here were all observed at similar hemispheric season; i.e., the crossings in both hemispheres experience a similar “effective” dipole tilt in both hemispheres. The observations of the northern hemisphere took place when the dipole was tilted away from the Sun by ∼10–14° in the northern hemisphere. The observations in the southern hemisphere took place 5–6 years later when the dipole was similarly tilted away from the Sun in the southern hemisphere by ∼14–17°. As these observations are in opposite hemispheres, a similar effective dipole tilt is experienced and, hence, no north‐south asymmetry would be expected. Indeed, when the fitting is repeated for crossings in the north and south separately allowing only E to vary, the same degree of flattening is found within the uncertainties. Hence, for this data set, seasonal variability cannot be quantified through modification of the functional form of the empirical model as was done in section 3.1.

However, seasonal variations may be taken into account using the “general deformation method” proposed by *Tsyganenko* [1998], who described how one can warp the current sheet and, indeed, the entire magnetopause surface in response to a dipole tilt. Such an effect has been observed in Saturn's current sheet by *Arridge et al.* [2008]. The form that *Tsyganenko* [1998] employed satisfies the condition that the current sheet follows the magnetic equator close to the planet but, at a distance known as the “hinging distance,” *R*_H_, the current sheet is gradually deflected out of the magnetic equator and is aligned parallel to solar wind flow at distances much larger than *R*_H_.

*Tsyganenko* [1998] described a procedure whereby one can warp a north‐south symmetric surface in Cartesian coordinates using his equations (7)–(11). Here this operation is performed in reverse by assuming that the magnetopause was warped when Cassini made its observations. The coordinates of the magnetopause crossings are hence points situated on this warped surface and can be transformed into the north‐south symmetric “dewarped,” or equinoctial, frame of reference described by equations (1), (2), (3) through simple algebraic manipulation to yield,

(6) $$\left(\begin{array}{c} X\\ Y\\ Z \end{array}\right)=\left(\begin{array}{ccc} \sin\lambda^{*} & 0 & \sin\lambda^{*}\\ 0 & 1 & 0\\ -\tan\lambda^{*} & 0 & \sec\lambda^{*} \end{array}\right)\left(\begin{array}{c} X^{*}\\ Y^{*}\\ Z^{*} \end{array}\right)$$

where * denotes coordinates in the warped frame (the crossing locations), coordinates without superscripts are in the dewarped frame and *λ* ^∗^ is the effective tilt angle, which is radially dependent and given by,

(7) $$\sin\lambda^{*}=\frac{R_{H}\sin(-\lambda )}{{\left(R_{H}^{3}+r^{3}\right)}^{1/3}}$$

where *r* is the planet‐crossing distance and *λ* is the dipole tilt as defined above. Note that the change in notation from *Tsyganenko* [1998] remains consistent with previous studies of the Kronian magnetopause. A first‐order approximation for the hinging distance, *R*_*H*_, is the standoff distance of the surface that passes directly through the crossing [*Arridge et al.*, 2008]. Using equation (6), the seasonal warping of the magnetopause can be removed from the observations and the empirical surface can then be fitted. Provided this is a satisfactory correction for the seasonal distortion of the magnetopause surface, the best fitting value of E is then the degree of polar flattening intrinsic to the shape of the obstacle.

**Figure 3:**
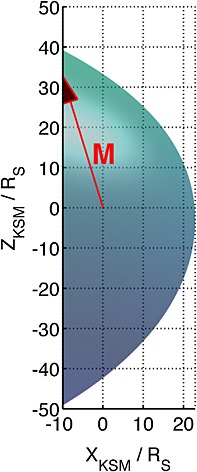
The warped magnetopause is shown with the dipole tilted 17° away from the Sun in the northern hemisphere. This introduces a clear north‐south asymmetry in its extent.

Fitting with this transformation reduced the RMS residual by ∼5% and yielded a polar flattening of 14 ± 1%, which is significantly different from the 19 ± 1% determined by *Pilkington et al.* [2015] who fitted the empirical model in the KSM reference frame in which the magnetopause geometry is subject to seasonal variability. This leads us to conclude that the presence of the magnetodisc imparts a polar flattening of 14 ± 1%. When the dipole is tilted 10–17° away from the Sun in either hemisphere, the surface is flattened by a further 5 ± 1% (providing a total flattening of 19 ± 1%) in that hemisphere and is presumably inflated in the opposite hemisphere. A representation of this modification is shown in Figure 3 though the magnetopause is likely to be warped even further under true solstice conditions. The warping of the surface probably varies monotonically with the degree of tilt and the asymmetries are likely to be present, though smaller, for tilts less than those considered here. This is supported by the fact that repeating the fitting after removing the warping using the procedure outlined above provides a better fit to the data.

## 4 Summary

Magnetometer and plasma data have been analyzed to construct an extended set of magnetopause crossings comprising coverage over most regions of Saturn's dayside magnetopause. These have been used to probe the structure of Saturn's magnetopause in greater detail than ever before.

This analysis has revealed a significant dawn‐dusk asymmetry in the size of the magnetosphere, with the magnetopause extending farther from the planet on the dawnside. It is suggested that this is caused by the intrinsic asymmetry in plasma flow around the planet with respect to the direction of solar wind flow. *Kivelson and Jia* [2014] have derived a similar, though larger, average asymmetry from MHD simulations. However, an opposing asymmetry in the suprathermal plasma *β* was also observed adjacent to the magnetopause, which likely acts to reduce the extent of the size asymmetry as the magnetopause can be perturbed more by internal plasma pressure on the duskside of the planet. This additional asymmetry may account for the discrepancy between the two results since the MHD simulation does not include a suprathermal plasma population.

In addition, since these measurements span a wide range of planetary season, it has been ascertained that a north‐south asymmetry is introduced during phases of the planetary season away from equinox, in agreement with theoretical work. This can be interpreted as an additional component of polar flattening and inflation in opposite hemispheres in addition to the intrinsic flattening due to the presence of the magnetodisc. These contributions have been separated using the general deformation method of *Tsyganenko* [1998], and a flattening intrinsic to the shape of the obstacle of 14 ± 1% has been found. This means that the remaining 5 ± 1% determined by *Pilkington et al.* [2014] and *Pilkington et al.* [2015] can be attributed to seasonal effects since their high‐latitude observations of the magnetopause occurred under conditions where additional seasonal confinement of the polar region is expected. High‐latitude observations of the magnetopause at solstice have been unavailable thus far, but it is likely that the magnetopause will be warped even further under solstice conditions. Observations over a wider range of season than are currently available would be useful in order to verify this and may mean that this effect could be incorporated into empirical models.

## References

[grl53350-bib-0001] Achilleos, N. , C. S. Arridge , C. Bertucci , C. M. Jackman , M. K. Dougherty , K. K. Khurana , and C. T. Russell (2008), Large‐scale dynamics of Saturn's magnetopause: Observations by Cassini, J. Geophys. Res., 113, A11209, doi:10.1029/2008JA013265.

[grl53350-bib-0002] Arridge, C. S. , N. Achilleos , M. K. Dougherty , K. K. Khurana , and C. T. Russell (2006), Modeling the size and shape of Saturn's magnetopause with variable dynamic pressure, J. Geophys. Res., 111, A11227, doi:10.1029/2005JA011574.

[grl53350-bib-0003] Arridge, C. S. , C. T. Russell , K. K. Khurana , N. Achilleos , N. André , a. M. Rymer , M. K. Dougherty , and A. J. Coates (2007), Mass of Saturn's magnetodisc: Cassini observations, Geophys. Res. Lett., 34, L09108, doi:10.1029/2006GL028921.

[grl53350-bib-0004] Arridge, C. S. , K. K. Khurana , C. T. Russell , D. J. Southwood , N. Achilleos , M. K. Dougherty , A. J. Coates , and H. K. Leinweber (2008), Warping of Saturn's magnetospheric and magnetotail current sheets, J. Geophys. Res., 113, A08217, doi:10.1029/2007JA012963.

[grl53350-bib-0005] Delamere, P. a. , and F. Bagenal (2013), Magnetotail structure of the giant magnetospheres: Implications of the viscous interaction with the solar wind, J. Geophys. Res. Space Physics, 118, 7045–7053, doi:10.1002/2013JA019179.

[grl53350-bib-0006] Dougherty, M. K. , et al. (2002), The Cassini magnetic field investigation, Space Sci. Rev., 114, 331–383.

[grl53350-bib-0007] Dougherty, M. K. , K. K. Khurana , F. M. Neubauer , C. T. Russell , J. Saur , J. S. Leisner , and M. E. Burton (2006), Identification of a dynamic atmosphere at Enceladus with the Cassini magnetometer, Science, 311(5766), 1406–1409, doi:10.1126/science.1120985. 16527966 10.1126/science.1120985

[grl53350-bib-0008] Hansen, K. C. , A. J. Ridley , G. B. Hospodarsky , N. Achilleos , M. K. Dougherty , T. I. Gombosi , and G. Toth (2005), Global MHD simulations of Saturn's magnetosphere at the time of Cassini approach, Geophys. Res. Lett., 32, L20S06, doi:10.1029/2005GL022835.

[grl53350-bib-0009] Joy, S. P. , M. G. Kivelson , R. J. Walker , K. K. Khurana , C. T. Russell , and T. Ogino (2002), Probabilistic models of the Jovian magnetopause and bow shock locations, J. Geophys. Res., 107(A10), 1309, doi:10.1029/2001JA009146.

[grl53350-bib-0010] Kanani, S. J. , et al. (2010), A new form of Saturn's magnetopause using a dynamic pressure balance model, based on in situ, multi‐instrument Cassini measurements, J. Geophys. Res., 115, A06207, doi:10.1029/2009JA014262.

[grl53350-bib-0011] Khurana, K. K. , and H. K. Schwarzl (2005), Global structure of Jupiter's magnetospheric current sheet, J. Geophys. Res., 110, A07227, doi:10.1029/2004JA010757.

[grl53350-bib-0012] Kivelson, M. G. , and X. Jia (2014), Control of periodic variations in Saturn's magnetosphere by compressional waves, J. Geophys. Res. Space Physics, 119, 8030–8045, doi:10.1002/2014JA020258.

[grl53350-bib-0013] Kivelson, M. G. , and D. J. Southwood (2005), Dynamical consequences of two modes of centrifugal instability in Jupiter's outer magnetosphere, J. Geophys. Res., 110, A12209, doi:10.1029/2005JA011176.

[grl53350-bib-0014] Krimigis, S. M. , T. P. Armstrong , W. I. Axford , C. O. Bostrom , G. Gloeckler , E. P. Keath , L. J. Lanzerotti , J. Carbary , D. C. Hamilton , and E. C. Roelof (1982), Low‐energy hot plasma and particles in Saturn's magnetosphere, Science, 215(4532), 571–577, doi:10.1038/098448b0. 17771280 10.1126/science.215.4532.571

[grl53350-bib-0015] Krimigis, S. M. , et al. (2004), Magnetosphere imaging instrument (MIMI) on the Cassini mission to Saturn/Titan, Space Sci. Rev., 114, 233–329.

[grl53350-bib-0016] Krimigis, S. M. , et al. (2005), Dynamics of Saturn's magnetosphere from MIMI during Cassini's orbital insertion, Science, 307(5713), 1270–1273, doi:10.1126/science.1105978. 15731445 10.1126/science.1105978

[grl53350-bib-0017] Krimigis, S. M. , N. Sergis , D. G. Mitchell , D. C. Hamilton , and N. Krupp (2007), A dynamic, rotating ring current around Saturn, Nature, 450(7172), 1050–1053, doi:10.1038/nature06425. 18075586 10.1038/nature06425

[grl53350-bib-0018] Massey, F. J. J. (1951), The Kolmogorov‐Smirnov test for goodness of fit, J. Am. Stat. Assoc., 46(253), 68–78.

[grl53350-bib-0019] Maurice, S. , I. M. Engle , M. Blanc , and M. Skubis (1996), Geometry of Saturn's magnetopause model, J. Geophys. Res., 101(A12), 27,053–27,059, doi:10.1029/96JA02605.

[grl53350-bib-0020] Petrinec, S. M. , and C. T. Russell (1995), An examination of the effect of dipole tilt angle and cusp regions on the shape of the dayside magnetopause, J. Geophys. Res., 100(A6), 9559–9566.

[grl53350-bib-0021] Petrinec, S. M. , and C. T. Russell (1997), Hydrodynamic and MHD equations across the bow shock and along the surfaces of planetary obstacles, Space Sci. Rev., 79, 757–791, doi:10.1023/A:1004938724300.

[grl53350-bib-0022] Pilkington, N. M. , N. Achilleos , C. S. Arridge , A. Masters , N. Sergis , A. J. Coates , and M. K. Dougherty (2014), Polar confinement of Saturn's magnetosphere revealed by in situ Cassini observations, J. Geophys. Res. Space Physics, 119, 2858–2875, doi:10.1002/2014JA019774.

[grl53350-bib-0023] Pilkington, N. M. , N. Achilleos , C. S. Arridge , P. Guio , A. Masters , L. C. Ray , N. Sergis , M. F. Thomsen , A. J. Coates , and M. K. Dougherty (2015), Internally driven large‐scale changes in the size of Saturn's magnetosphere, J. Geophys. Res. Space Physics, 120, doi:10.1002/2015JA021290. 10.1002/2015JA021290 PMC5111417 27867793

[grl53350-bib-0024] Porco, C. C. , et al. (2006), Cassini observes the active south pole of Enceladus, Science, 311, 1393–1401, doi:10.1126/science.1123013. 16527964 10.1126/science.1123013

[grl53350-bib-0025] Sergis, N. , S. M. Krimigis , D. G. Mitchell , D. C. Hamilton , N. Krupp , B. H. Mauk , E. C. Roelof , and M. K. Dougherty (2009), Energetic particle pressure in Saturn's magnetosphere measured with the Magnetospheric Imaging Instrument on Cassini, J. Geophys. Res., 114, A02214, doi:10.1029/2008JA013774.

[grl53350-bib-0026] Shue, J. H. , J. K. Chao , H. C. Fu , C. T. Russell , P. Song , K. K. Khurana , and H. J. Singer (1997), A new functional form to study the solar wind control of the magnetopause size and shape, J. Geophys. Res., 102(A5), 9497–9511, doi:10.1029/97JA00196.

[grl53350-bib-0027] Slavin, J. A. , E. J. Smith , P. R. Gazis , and J. D. Mihalov (1983), A pioneer‐voyager study of the solar wind interaction with Saturn, Geophys. Res. Lett., 10(1), 9–12.

[grl53350-bib-0028] Tsyganenko, N. A. (1998), Modeling of twisted/warped magnetospheric configurations using the general deformation method, J. Geophys. Res., 103(A10), 23,551–23,563, doi:10.1029/98JA02292.

[grl53350-bib-0029] Ugray, Z. , L. Lasdon , J. Plummer , F. Glover , J. Kelly , and R. Marti (2007), Scatter search and local NLP solvers: A multistart framework for global optimization, INFORMS J. Comput., 19(3), 328–40.

[grl53350-bib-0030] Waltz, R. , J. Morales , J. Nocedal , and D. Orban (2005), An interior algorithm for nonlinear optimization that combines line search and trust region steps, Math. Program., 107(3), 391–408, doi:10.1007/s10107‐004‐0560‐5.

[grl53350-bib-0031] Young, D. T. , et al. (2004), Cassini plasma spectrometer investigation, Space Sci. Rev., 114, 1–112.

